# TNF Patterns and Tumor Microenvironment Characterization in Head and Neck Squamous Cell Carcinoma

**DOI:** 10.3389/fimmu.2021.754818

**Published:** 2021-10-06

**Authors:** Qian Long, Chunyu Huang, Qi Meng, Jin Peng, Fan Yao, Dingfu Du, Xiaonan Wang, Wancui Zhu, Dingbo Shi, Xiangdong Xu, Xiang Qi, Wuguo Deng, Miao Chen, Yizhuo Li, Ankui Yang

**Affiliations:** ^1^ Sun Yat-sen University Cancer Center, State Key Laboratory of Oncology in South China, Collaborative Innovation Center of Cancer Medicine, Guangzhou, China; ^2^ The First Affiliated Hospital of Sun Yat-sen University, Guangzhou, China; ^3^ Department of Scientific Research, Guangdong Sanqiantang Medical Research Institute, Guangzhou, China

**Keywords:** TNF, head and neck cancer, tumor immune microenvironment, immunotherapy, bioinformatics and biomarkers

## Abstract

**Background:**

HNSCC is a heterogeneous disease, which arises from distinct anatomic subsites, associates with various risk factors and possesses diverse molecular pathological features. Generally, HNSCC is considered as an immunosuppressive disease, characterized by abnormal tumor immune microenvironment. The TNF family plays a crucial role in the survival, proliferation, differentiation, and effector functions in both immune and non-immune cells. However, the expression patterns of TNF in HNSCC remains to be systematically analyzed.

**Methods:**

We downloaded transcriptional profile data of HNSCC from TCGA and GEO datasets. Unsupervised clustering methods were used to identify different TNF patterns and classify patients for further analysis. PCA was conducted to construct a TNF relevant score, which we called risk score.

**Results:**

In this study, we systematically evaluated the patterns of TNF family and tumor immune microenvironment characteristics of HNSCC patients by clustering the expression of 46 members of TNF family. We identified two subtypes with distinct clinical and immune characteristics in HNSCC and constructed a risk scoring system based on the expression profile of TNF family genes.

**Conclusion:**

Risk score serves as a reliable predictor of overall survival, clinical characteristics, and immune cell infiltration, which has the potential to be applied as a valuable biomarker for HNSCC immunotherapy.

## Introduction

Head and neck squamous cell carcinoma (HNSCC) is one of the most common malignant tumors worldwide (1, 2). HNSCC is a heterogeneous disease, which arises from distinct anatomic subsites, associates with various risk factors and possesses diverse molecular pathological features (3). Generally, HNSCC is considered as an immunosuppressive disease, characterized by abnormal tumor immune microenvironment (4). Two immune checkpoint inhibitors, pembrolizumab and nivolumab have been approved for the treatment of advanced HNSCC by FDA (5, 6), but only a limited number of patients with HNSCC benefit from immune checkpoint inhibitors. It is therefore urgent to identify reliable molecular biomarkers for risk stratification and therapeutic benefits prediction for immunotherapies in HNSCC (4).

The TNF family, which consists of a 19 TNF ligand superfamily (TNFSF) and a 29 TNF receptor superfamily (TNFRSF), plays a crucial role in the survival, proliferation, differentiation, and effector functions in both immune and non-immune cells (7). A number of TNF family members have been verified to be associated with human diseases including inflammatory disease and cancer (8). Because of the vital role TNF family activities in inflammatory responses regulation, antagonists targeting this signaling to reduce chronic inflammation or promote anti-tumor immunity have been developed, and tested or being tested in clinical trials for inflammatory diseases or cancer (9). In the context of head and neck cancer, TNF signaling was a well-established tumor-promoting pathway *via* either helping tumor cell resist apoptosis or inducing an immune suppressive tumor microenvironment (10–14). For instance, OX40, a member of the TNFRSF, was reported to be highly expressed in the tumor infiltrating lymphocytes of patients with HNSCC, leading to suppressive tumor immune microenvironment (10). Another study demonstrated that TNF-α promotes invasion and metastasis *via* NF-κB pathway in oral squamous cell carcinoma (15). However, the expression patterns and functions of TNF family in HNSCC remains to be systematically analyzed.

In this study, we systematically evaluated the patterns of TNF family and tumor immune microenvironment characteristics of HNSCC patients by clustering the expression of 46 members of TNF family. We identified two subtypes with distinct clinical and immune characteristics in HNSCC and constructed a risk scoring system based on the expression profile of TNF family genes. Risk score serves as a reliable predictor of overall survival, clinical characteristics, and immune cell infiltration, which has the potential to be applied as a valuable biomarker for HNSCC immunotherapy.

## Materials and Methods

### Data and Resources

All data used in this study were obtained from public databases. In total, three HNSCC cohorts were included in our study (TCGA, GSE65858 and GSE41613). For TCGA HNSCC cohort, RNA sequencing data [fragments per kilobase of transcript per million mapped reads (FPKM) values] were downloaded *via* the R package TCGAbiolinks (16). Two GEO datasets were downloaded and processed by R package GEOquery. Then, FPKM values were transformed into transcripts per kilobase million (TPM) values that were more similar to those generated from microarrays. Somatic mutation (SNPs and small INDELs) was downloaded from the University of California Santa Cruz (UCSC) Xena browser (https://xenabrowser.net). All baseline information of HNSCC datasets is summarized in **Supplementary Table S1**.

### Unsupervised Clustering for TNF Family Genes

Unsupervised clustering methods were used to identify different TNF patterns and classify patients for further analysis. A total of 46 TNF family genes were used to conduct the unsupervised clustering. A consensus clustering algorithm was performed using the R package ConsensuClusterPlus (17) and was repeated 1,000 times in order to ensure the stability of clustering. The group information after unsupervised clustering of TCGA HNSCC cohort is in **Supplementary Table S2**.

### Gene Set Variation Analysis (GSVA) and Single-Sample GSEA (ssGSEA)

The R package GSVA (18) was used to quantify the activity of biological pathways. Immune gene signatures were collected from previously published works (19) (**Supplementary Table S3**). The relative immune signature enrichment scores of each TCGA HNSCC sample are in **Supplementary Table S4**. The ssGSEA algorithm in the R package GSVA was used to estimate the relative abundance of each immune cell in HNSCC. The gene sets defining each immune cell type were downloaded from the study of Charoentong (20) (**Supplementary Table S5**). The relative abundance of each immune cell of each TCGA HNSCC sample was supplemented in **Supplementary Table S6**.

### The Protein-Protein Interactions (PPI) Analysis

The protein-protein interactions among TNF family proteins were identified on the STRING according to the instructions (21).

### Functional and Pathway Enrichment Analysis

GO analysis was performed to identify enriched GO terms using the R package clusterProfiler (22) with a cutoff *p* value <0.05 and an adjusted *p* value <0.2. To identify the most related pathways of TNF family genes, the gseKEGG function of the R package clusterProfiler (22) was used. The DEGs list was estimated between groups with high and low expression of this gene and ranked according to adjusted *p* value.

### DEGs Among TNF Patterns

DEGs among two TNF patterns (**Supplementary Table S7**) were determined using the R package limma (23). The significance criterion for DEGs was set as an adjusted *p* value < 0.001 and log FC > 1 or < -1.

### Generation of the Risk Score

First, the prognostic analysis was performed for each gene in the 693 DEGs using a univariate Cox regression model. A total of 177 genes (**Supplementary Table S8**) with significant prognosis were extracted for further analysis. Then, the expression of these genes was transformed into a Z score. PCA was conducted to construct a TNF relevant score, which we called risk score. Both PC1 and PC2 were selected to serve as signature scores. Risk score = ∑(PC1_i_ + PC2_i_), where i is the expression of TNF family pattern related signature genes. Risk score of TCGA and two independent GEO cohorts are in **Supplementary Table S9**.

### Statistical Analysis

The normality of the variables was tested using the Shapiro-Wilk normality test (24). For comparisons of two normally distributed groups, statistical analysis was performed by unpaired t tests, and for nonnormally distributed variables, statistical analysis was analyzed by a Wilcoxon rank-sum test. The best cutoff values of each cohort were evaluated using the survcutpoint function in the survminer package. The survival curves for the prognostic analysis were conducted *via* the Kaplan-Meier method, and log-rank tests were utilized to judge differences between groups. Correlation coefficients were computed by Spearman and distance correlation analyses. The univariate Cox regression model was utilized to calculate the hazard ratios (HRs). All statistical *p* values were two-tailed, with *p <*0.05 considered as statistically significant. All statistical analyses were conducted using R 4.0.5 (https://www.r-project.org/). (* *p* < 0.05, ** *p* < 0.01, *** *p* < 0.001, **** *p* < 0.0001).

## Results

### Multi-Omics Landscape of TNF Family in HNSCC

**Figure 1:**
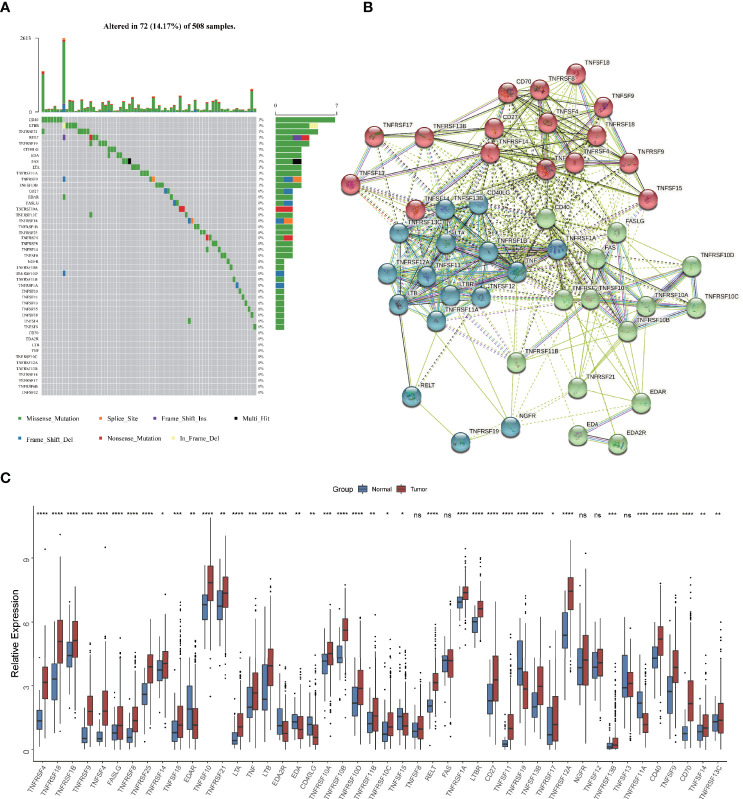
Multi-omics landscape of TNF family in HNSCC. **(A)** The mutation frequency of 46 TNF family proteins in TCGA HNSCC cohort. Each column of the figure represents an individual patient. **(B)** The protein-protein interaction network among TNF family proteins. **(C)** Boxplot shows the expression of 46 TNF family proteins between tumor and normal tissues in TCGA HNSCC cohort. (*p < 0.05, **p < 0.01, ***p < 0.001, ****p < 0.0001, ns, not significant)

Firstly, we depicted the multi-omics landscape of 46 TNF family proteins using data from TCGA HNSCC cohort (TNFRSF6B was excluded because of its undetectable expression in TCGA HNSC cohort). We found that the overall mutation rate of all proteins is relatively low in the HNSCC genome (**Figure 1A**). Next, the protein-protein interaction (PPI) network analyzed by STRING showed that TNFSF and TNFRSF had widespread protein interactions, and members inside TNFSF or TNFRSF also interacted extensively (**Figure 1B**). These results demonstrated that members among TNF family formed a complicated network to synergistically mediate TNF pathway in tumor progression. As shown in **Figure 1C**, the expression of most proteins was significantly elevated in tumor tissues compared to normal adjacent tissues.

### The Immune Correlation and Prognostic Value of TNF Family in HNSCC

**Figure 2:**
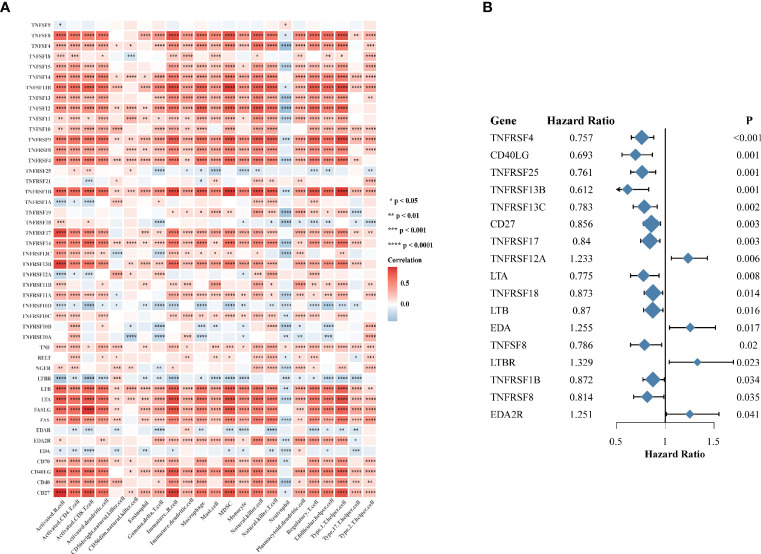
The immune correlation and prognostic value of TNF family in HNSCC. **(A)** Correlation heatmap between 46 TNF family proteins and immune cells in the TCGA HNSCC cohort. Orange indicates positive correlation; blue indicates negative correlation. **(B)** The forest plot of the univariate Cox regression model depicting the 17 statistically significant prognostic factors in TNF family proteins in TCGA HNSCC cohort. Hazard ratio > 1: risk factors for survival. Hazard ratio < 1: protective factors for survival. (*p < 0.05, **p < 0.01, ***p < 0.001, ****p < 0.0001).

Considering the vital roles of TNF family in immune regulation (7), we speculated that they might be associated with immune cell infiltration in tumor microenvironment in HNSCC. We analyzed the association between expression of 46 TNF family proteins with 23 types of immune cells in TCGA HNSCC cohort respectively, and we found that most of them were significantly positively correlated with immune cell infiltration in tumor tissues (**Figure 2A**). To clarify the prognostic significance of TNF family, we conducted univariate Cox regression of all 46 proteins, and found that 17 proteins were significantly correlated with overall survival of HNSCC patients, of which only TNFRSF12A, EDA, LTBR and EDA2R predicted unfavorable outcomes (**Figure 2B**). These results seemed contradictory to our previous result that most TNF family members were elevated in tumor tissues compared to normal adjacent tissues. However, patients enrolled from TCGA HNSCC cohort have received anti-tumor therapies, and these results indicated that high expression of TNF family members could have better therapeutic responses, thus leading to a better overall survival.

### TNF Patterns in the TCGA HNSCC Cohort

**Figure 3:**
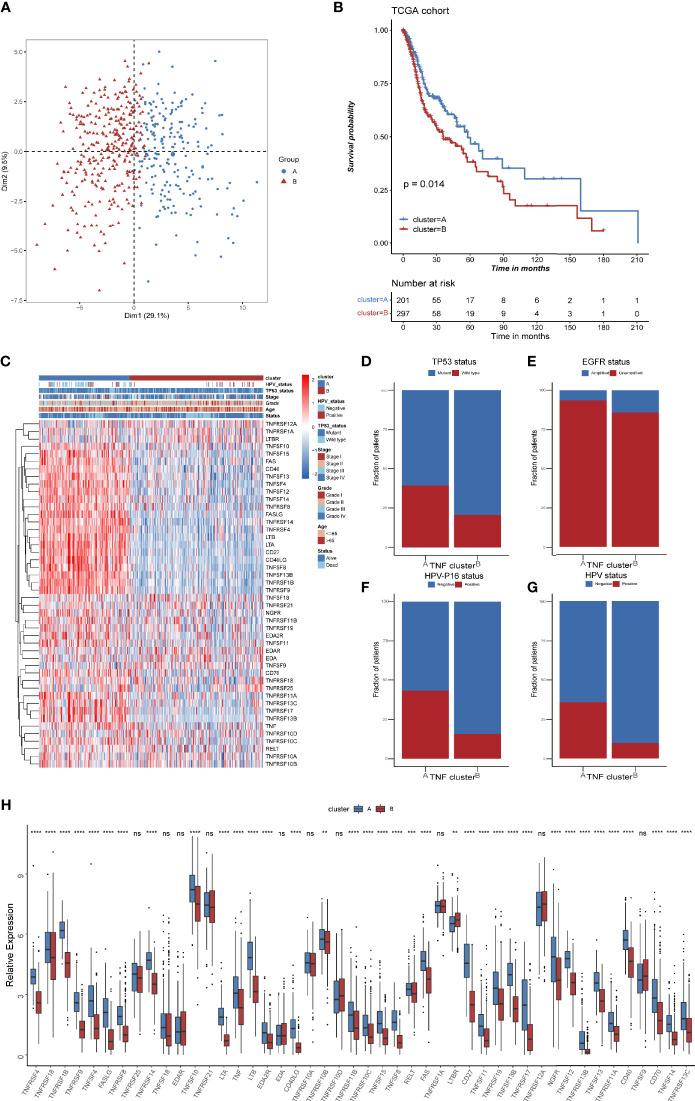
TNF patterns in the TCGA HNSCC cohort. **(A)** Principal component analysis (PCA) for the expression of 46 TNF/TNFR proteins to distinguish two TNF/TNRER patterns in TCGA HSNC cohort. **(B)** Kaplan-Meier survival analysis of two TNF patterns in TCGA HNSCC cohort. **(C)** Unsupervised clustering of 46 TNF family proteins in TCGA HNSCC cohort. Red represents high expression, and blue represents low expression. The TNF patterns, HPV status, TP53 status, stage, pathological grade, age, and survival status were used as sample annotations. **(D–G)** Stacked bar plot of TCGA HNSCC TP53 status, HPV-16 status, HPV status and EGFR status in two TNF patterns, respectively. **(H)** Relative expression of 46 TNF family proteins in TNF cluster A and B in TCGA HNSCC cohort. (**p < 0.01, ***p < 0.001, ****p < 0.0001, ns, not significant).

TNF family might contribute to the heterogeneity of HNSCC and they were also associated closely with the tumor immune microenvironment. To further identify new probable TNF family patterns, unsupervised clustering was conducted based on the expression of 46 members of TNF family in TCGA HNSCC cohort. As shown in **Supplementary Figures S1A–D**, two clusters could achieve the best clustering efficacy. Accordingly, patients were classified into TNF pattern A and pattern B (**Figure 3A**). TNF pattern A displayed better overall survival, whereas TNF pattern B displayed more advanced pathological stage and grade (**Figures 3B, C**). More elaborate analysis of TNF patterns with clinical characteristics of HNSCC patients showed that TNF pattern A had a low frequency of TP53 mutation and EGFR amplification (**Figures 3D, E**), but a higher rate of HPV infection (**Figures 3F, G**). TP53 mutation and EGFR amplification are unfavorable predictors in HNSCC patients, while HPV infection was a favorable indicator. These results were consistent with TNF pattern A with a better overall survival in HNSCC. Furthermore, almost all TNF family proteins were significantly elevated in TNF pattern A (**Figure 3H**).

### Differential Immune Characteristics of TNF Pattern A and B

**Figure 4:**
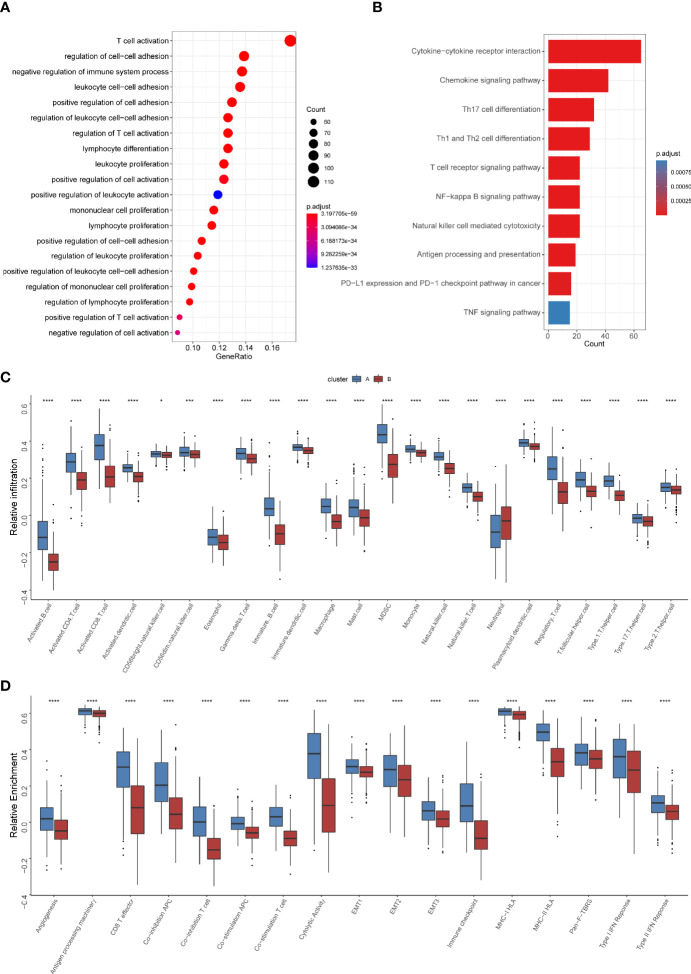
Differential immune characteristics of TNF pattern A and B **(A)** Gene Ontology (GO) analysis depicted the enriched pathways of TNF-related genes. **(B)** Kyoto Encyclopedia of Genes and Genomes (KEGG) pathways analysis of TNF-related genes. **(C)** Relative infiltration of 23 types of immune cells in TNF cluster A and B **(D)** Relative enrichment score of 17 immune related signatures in TNF cluster A and B. (*p < 0.05, ***p < 0.001, ****p < 0.0001).

To further explore the heterogeneity of different TNF patterns, we identified the differentially expressed genes (DEGs) among two groups. A total of 693 TNF pattern-related genes were identified (**Supplementary Table S7**). Gene Ontology (GO) and Kyoto Encyclopedia of Genes and Genomes (KEGG) analysis showed that the pathways were enriched in immune-related events (**Figures 4A, B**). Also, we conducted gene set enrichment analysis (GSEA) of differentially expressed genes between TNF pattern A and B, and the result showed that they are enriched in antigen processing and presentation and T cell differentiation (**Supplementary Figure S2**), which was consistent with our GO and KEGG analysis. To further analyze immune cell infiltration and immune-related signatures in two TNF patterns, we estimated the relative abundance of each type of immune cells and relative immune signature enrichment scores in HNSCC by the method of single-sample GSEA (ssGSEA). The results showed that TNF pattern A was significantly associated with immune cell infiltration (especially CD4+ and CD8+ T cell) and relatively hot immune microenvironment signature, such as effector CD8+ T cells and cytolytic activity (**Figures 4C, D**). Considering the vital role of immune checkpoint molecules (PD-1, PD-L1, LAG3 and CTLA-4) in tumor immune microenvironment, we analyzed the expression of these molecules in two TNF patterns. We found that the expression of all four immune checkpoint molecules was significantly elevated in TNF pattern A, which was consistent with our above-mentioned immune-related signatures (**Supplementary Figure S3**).

### The Clinical and Transcriptomic Characteristics of TNF-Related Gene Clusters

**Figure 5:**
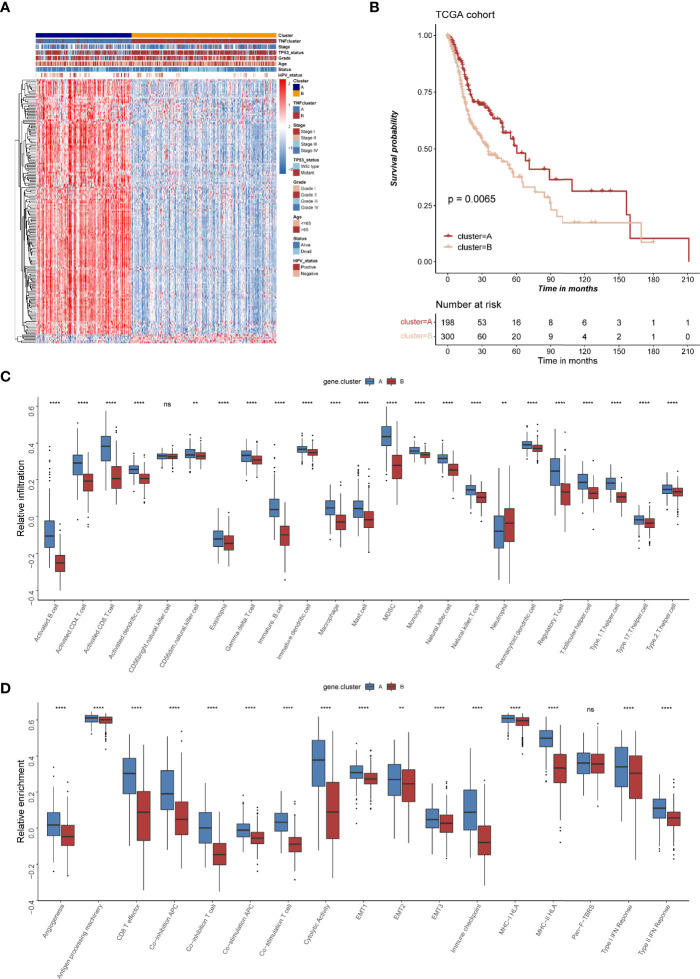
The clinical and transcriptomic characteristics of TNF-related gene clusters. **(A)** Unsupervised clustering of 177 TNF related genes in TCGA HNSCC cohort. Red represents high expression, and blue represents low expression. The TNF gene cluster patterns, TNF patterns, HPV status, TP53 status, stage, pathological grade, age, and survival status were used as sample annotations. **(B)** Kaplan-Meier survival analysis of two TNF gene cluster patterns in TCGA HNSCC cohort. **(C)** Relative infiltration of 23 types of immune cells in TNF gene cluster A and B **(D)** Relative enrichment score of 17 immune related signatures in TNF gene cluster A and B. ( **p < 0.01, ****p < 0.0001, ns, not significant).

To further explore the heterogeneity of different TNF patterns, a univariate Cox regression analysis of 693 DEGs certified that 177 genes had prognostic value (**Supplementary Table S8**). Unsupervised clustering analysis based on the expression of these 177 genes also divided HNSCC patients into two clusters, which we called TNF gene clusters (**Supplementary Figure S4**). The clinical analysis showed that different gene cluster had distinct status of TP53 mutation and HPV infection (**Figure 5A**), which was consistent with our previous identified TNF clusters, and TNF gene cluster A also tended to have a better survival (**Figure 5B**). Further analysis of two TNF gene clusters with immune cell infiltration and immune related signature also showed gene cluster A was enriched in immune cell infiltration and immune signatures (**Figures 5C, D**). Furthermore, we analyzed the expression of four important immune checkpoint molecules (PD-1, PD-L1, LAG3 and CTLA-4) in two TNF gene clusters, and the results showed that TNF gene cluster A showed a significant elevation of all four molecules, which was an indicator of hot immune microenvironment (**Supplementary Figure S5**). Additionally, the expression of most TNF family proteins was significantly elevated in TNF gene cluster A (**Supplementary Figure S6A**).

### The Construction of Risk Score and Its Clinical Significance

**Figure 6:**
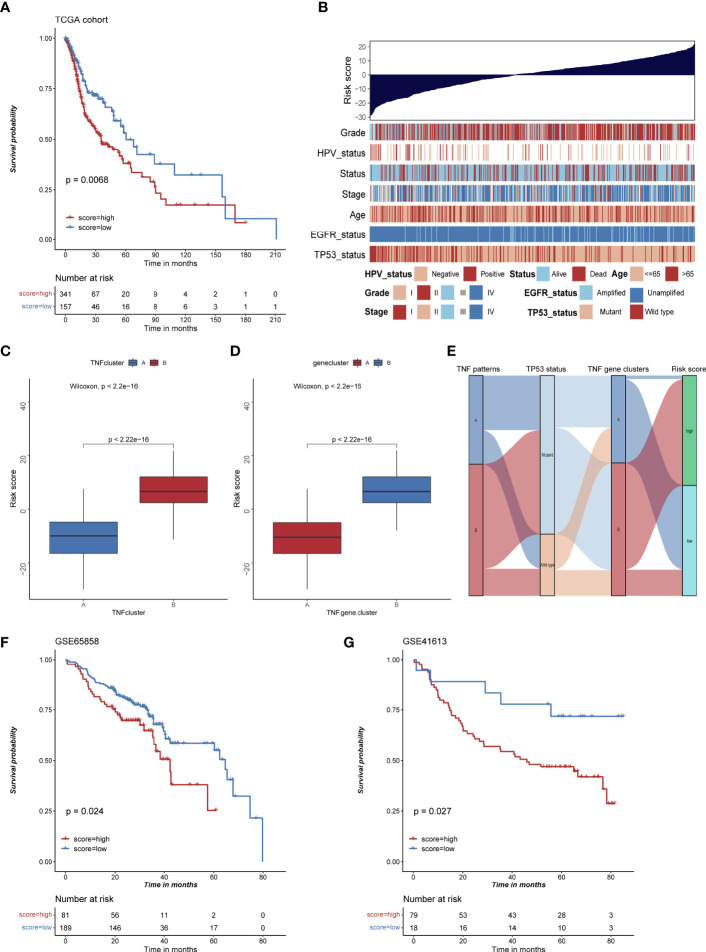
The construction of risk score and its clinical significances. **(A)** Kaplan-Meier survival analysis of high and low risk score group in TCGA HNSCC cohort. **(B)** Bar chart depicting the relationship of risk score and various clinical characteristics in the TCGA HNSCC cohort. HPV status, TP53 status, stage, pathological grade, age, and survival status were used as sample annotations. **(C)** Boxplot of risk score in TNF cluster A and B **(D)** Boxplot of risk score in TNF gene cluster A and B **(E)** Alluvial diagram depicting the relationship of TNF cluster, TNF gene cluster, TP53 status and risk score group, TNF cluster/gene cluster A was associated with low frequency of TP53 mutation and low risk score. **(F)** Kaplan-Meier survival analysis of high and low risk score group in GSE65858 cohort. **(G)** Kaplan-Meier survival analysis of high and low risk score group in GSE41613 cohort.

To evaluate TNF status individually, we further constructed a risk score system based on 177 TNF-related signature genes. High risk score was associated with worse outcome, high frequency of TP53 mutation and EGFR amplification, and low frequency of HPV infection in HNSCC patients (**Figures 6A, B**), whereas TNF pattern A and TNF gene cluster A, which had better overall survival, showed a low risk score (**Figures 6C–E**). Also, high risk score was correlated with less immune cell infiltration and lower immune signature enrichment (**Supplementary Figure S6A, B**). Moreover, risk score was significantly negatively correlated with the expression of immune checkpoint molecules (PD1, PDL1, LAG3 and CTLA4) (**Supplementary Figures S7A–D**). Subsequently, we collected two independent GEO cohorts to further validate prognostic values of our risk score. As shown in **Figures 6F, G**, high risk score was significantly correlated with poor overall survival in two independent cohorts, and TP53 mutation or HPV negative group tended to have high risk scores (**Supplementary Figure S8**).

## Discussion

HNSCC is considered as a disease with extraordinary heterogeneity due to its diverse anatomic subsites (1). Risk factors for this disease include tobacco consumption, alcohol abuse and infection with human papillomavirus (HPV), primarily HPV-16 (25–27). With the development of next-generation sequencing, substantial progress has been made over the past decade in the molecular characterization of HNSCC, culminating in The Cancer Genome Atlas (TCGA) (1, 28–30). Based on the multi-omics data from TCGA, a series of studies have identified a number of genes or signatures for risk stratification, prognosis prediction and therapeutic responses prediction. However, novel molecular biomarkers are still urgently to be identified for better management of this disease.

TNF signaling was a well-established tumor-promoting pathway by either helping tumor cells resist apoptosis or inducing an immune suppressive tumor microenvironment in HNSCC (10–14). We firstly systematically analyzed multi-omics data of 46 TNF family genes, and we found that most of these genes were significantly differentially expressed in HNSCC. Further analysis of immune correlation of TNF family genes in HNSCC revealed that most of them were significantly positively correlated with infiltration of various immune cells, including activated CD4^+^ and CD8^+^ T cell. Univariate Cox regression of all 46 proteins showed that 17 proteins were significantly correlated with overall survival of HNSCC patients, of which only TNFRSF12A, EDA, LTBR and EDA2R predicted unfavorable outcomes. These results demonstrated that TNF family genes played a crucial role in the development and TME of HNSCC.

Subsequently, we identified two subtypes with distinct clinical and immune characteristics in HNSCC based on the expression profile of TNF family genes. TNF cluster A was characterized by high infiltration of immune cells and enrichment of immune activated signatures along with a better overall survival. TP53 mutation (72% of tumors) was the most frequent mutation in HNSCC, and it was considered as an early driver genomic alterations (28). Our analysis found that TNF cluster A had lower TP53 mutation frequency compared to TNF cluster B. Another oncogenic driver gene in HNSCC tumor is EGFR, which was overexpressed in 80–90% of HNSCC tumors and was associated with poor overall survival and progression-free survival (31, 32). Our analysis of EGFR amplification in TNF cluster A and B also showed that cluster A had higher fraction of unamplified EGFR compared cluster B. HPV infection is an increasingly common risk factor for HNSCC. HPV infection is associated with most oropharyngeal cancers (>70%) and a small minority of cancers at other head and neck anatomical sites (25, 26), and HPV-positive HNSCC generally has a more favorable prognosis than HPV-negative HNSCC (1). We also analyzed the relationship between TNF clusters and HPV infection in HNSCC, and the result showed that HPV infection was more frequent in TNF cluster A. All these results were consistent with our previous analysis that TNF cluster A had a better overall survival.

The tumor microenvironment (TME) in HNSCC is a complex and heterogeneous population of tumor cells and stromal cells, which include endothelial cells, cancer-associated fibroblasts (CAFs) and immune cells (1). Generally, HNSCC is considered as an immune cell infiltrating tumor, which are characterized by immunosuppressive TME (33, 34). Two immune checkpoint inhibitors, pembrolizumab and nivolumab have been approved for the treatment of advanced HNSCC by FDA (5, 6), but a limited number of patients with HNSCC derive benefit from immune checkpoint inhibitors. It is also urgent to identify reliable molecular biomarkers for risk stratification and therapeutic benefit prediction for those therapy strategies in HNSCC (4). In our study, two subtypes identified by us had distinct immune characteristics. TNF cluster A was significantly associated with higher immune cell infiltration, activated immune signatures and expression of immune checkpoint molecules, which indicated an immune “hot” tumor. Based on this result, we speculate that TNF cluster A might derive better responses to immune checkpoint inhibitors.

Considering the distinct characteristics of two TNF clusters, we hypothesized that DEGs between two groups might also have unique characteristics. Accordingly, we conducted unsupervised clustering analysis based on the expression of DEGs and also divided HNSCC patients into two groups, which we called TNF gene cluster A and B. Similar to the results of TNF clusters, two TNF gene clusters also had distinct clinical and immune characteristics. These results demonstrated that there were indeed two immune subtypes based on the expression profile of TNF gene family in HNSCC.

Regarding the clinical significance of our study, we constructed a risk score based on the expression of DEGs that can evaluate risk of HNSCC patients individually. Indeed, the patients with high risk score had worse overall survival, which was validated by two independent GEO cohorts. Also, the patients with TP53 mutation or EGFR amplification had a significantly higher risk score and patients with HPV infection had a lower risk score, which was consistent with our TNF clusters and TNF gene clusters.

However, our study also had some limitations. First, we failed to find expression profile data of HNSCC patients receiving immune checkpoint blockers to validate the predictive value of our risk score to immune checkpoint inhibitors. Second, our analysis was only based on retrospective data from public databases, which need to be further validated by prospective studies in the future.

In conclusion, we identified two subtypes with distinct clinical and immune characteristics in HNSCC and constructed a risk scoring system based on the expression profile of TNF genes. Risk score is capable of serving as a reliable predictor of overall survival, clinical characteristics, and immune cell infiltration, which have the potential to be applied as a valuable biomarker for HNSCC immunotherapy.

## Data Availability Statement

The datasets presented in this study can be found in online repositories. The names of the repository/repositories and accession number(s) can be found in the article/**Supplementary Material**.

## Author Contributions

QL, YL, MC, and AY conceived and designed the project. QL, CH, QM, JP, WD, XX, DD, XW, XQ, WZ, DS, and MC analyzed and interpreted the data. QL, YL, MC, and AY wrote and revised the manuscript. All authors contributed to the article and approved the submitted version.

## Funding

This work was supported by the funds from the National Natural Science Foundation of China (81902681, 81972569, 81772925, 82072981, 81972623).

## Conflict of Interest

The authors declare that the research was conducted in the absence of any commercial or financial relationships that could be construed as a potential conflict of interest.

## Publisher’s Note

All claims expressed in this article are solely those of the authors and do not necessarily represent those of their affiliated organizations, or those of the publisher, the editors and the reviewers. Any product that may be evaluated in this article, or claim that may be made by its manufacturer, is not guaranteed or endorsed by the publisher.

## Supplementary Material

Supplementary Figure 1
**(A–D)** Consensus clustering matrices of 46 TNF family proteins in TCGA HNSCC cohort for k = 2-5.

Supplementary Figure 2 GSEA of DEGs in two TNF patterns in HNSCC.

Supplementary Figure 3 The relative expression of PD1, PDL1, LAG3 and CTLA4 in two TNF patterns in HNSCC.

Supplementary Figure 4
**(A–D)** Consensus clustering matrices of 177 differentially expressed genes in TCGA HNSCC cohort for k = 2-5.

Supplementary Figure 5 The relative expression of PD1, PDL1, LAG3 and CTLA4 in two TNF gene clusters in HNSCC.

Supplementary Figure 6
**(A)** Relative expression of 46 TNF family proteins in TNF cluster A and B in TCGA HNSCC cohort. **(B)** Relative infiltration of 23 types of immune cells in high and low risk group. **(C)** Relative enrichment score of 17 immune related signatures in high and low risk group.

Supplementary Figure 7
**(A–D)** The correlation of risk score with the expression of PD1, PDL1, LAG3 and CTLA4 in TCGA HNSCC cohort, respectively.

Supplementary Figure 8
**(A)** Risk score in different TP53 status patients in GSE65858 cohort. **(B)** Risk score in different HPV status patients in GSE65858 cohort.

## Abbreviations

HNSCC, head and neck squamous cell carcinomas; TNFSF, TNF ligands superfamily; TNFRSF, TNF receptor superfamily; GSVA, gene set variation analysis; PCA, Principal component analysis; ssGSEA, single-sample gene set enrichment analysis; PPI, protein-protein interactions; DEGs, differentially expressed genes; GO, Gene Ontology; KEGG, Kyoto Encyclopedia of Genes and Genomes; TME, tumor microenvironment; CAFs, cancer-associated fibroblasts; HPV, human papillomavirus.
