# Supplementary figures and images for: TNF Patterns and Tumor Microenvironment Characterization in Head and Neck Squamous Cell Carcinoma
Source: Front Immunol. 2021 Oct 6;12:754818. doi: 10.3389/fimmu.2021.754818 (PMC8526904; doi:10.3389/fimmu.2021.754818)

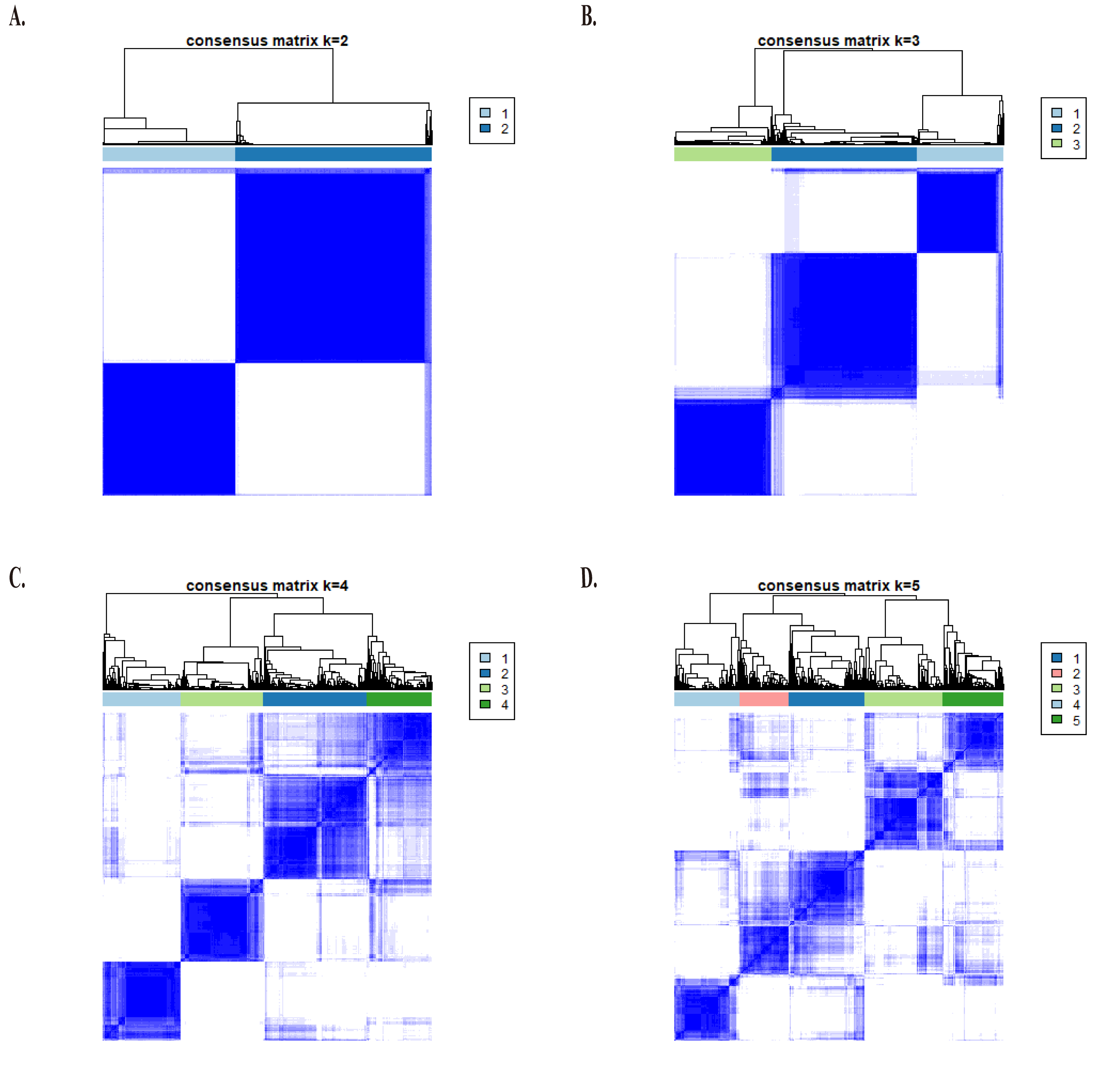

Supplement: Supplementary Figure 1 — (A–D) Consensus clustering matrices of 46 TNF family proteins in TCGA HNSCC cohort for k = 2-5. [file Image_1.tif]

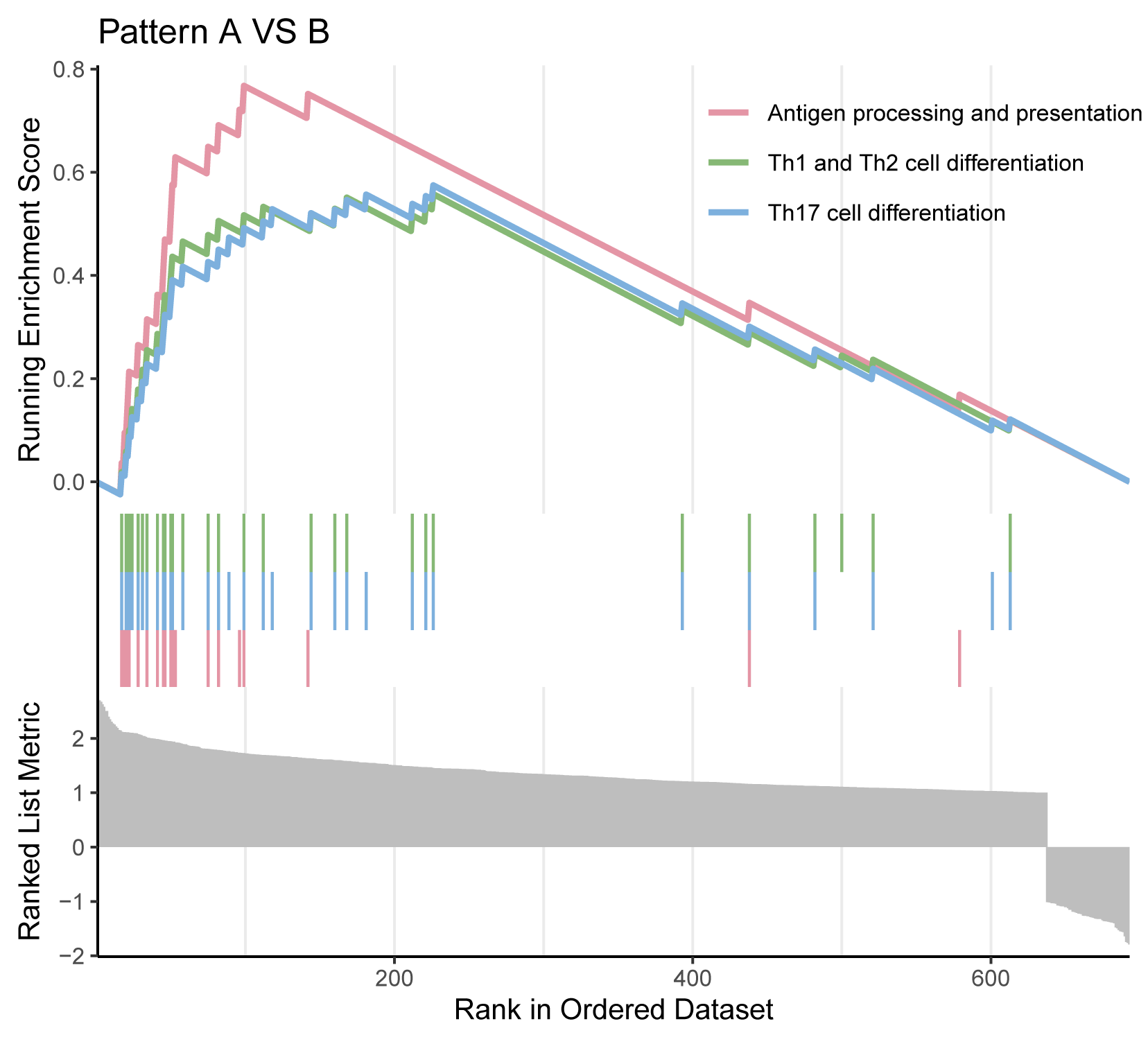

Supplement: Supplementary Figure 2 — GSEA of DEGs in two TNF patterns in HNSCC. [file Image_2.tif]

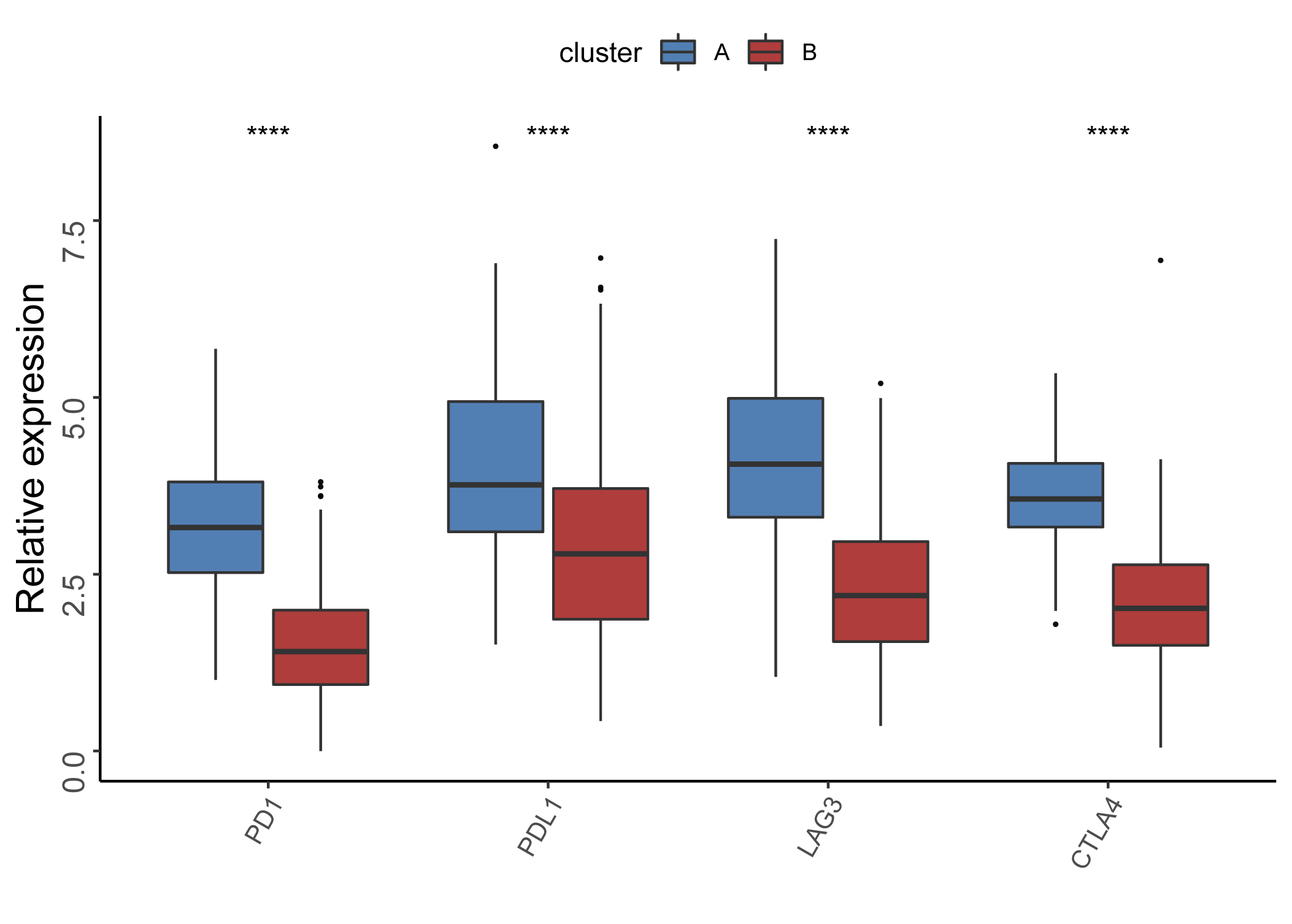

Supplement: Supplementary Figure 3 — The relative expression of PD1, PDL1, LAG3 and CTLA4 in two TNF patterns in HNSCC. [file Image_3.tif]

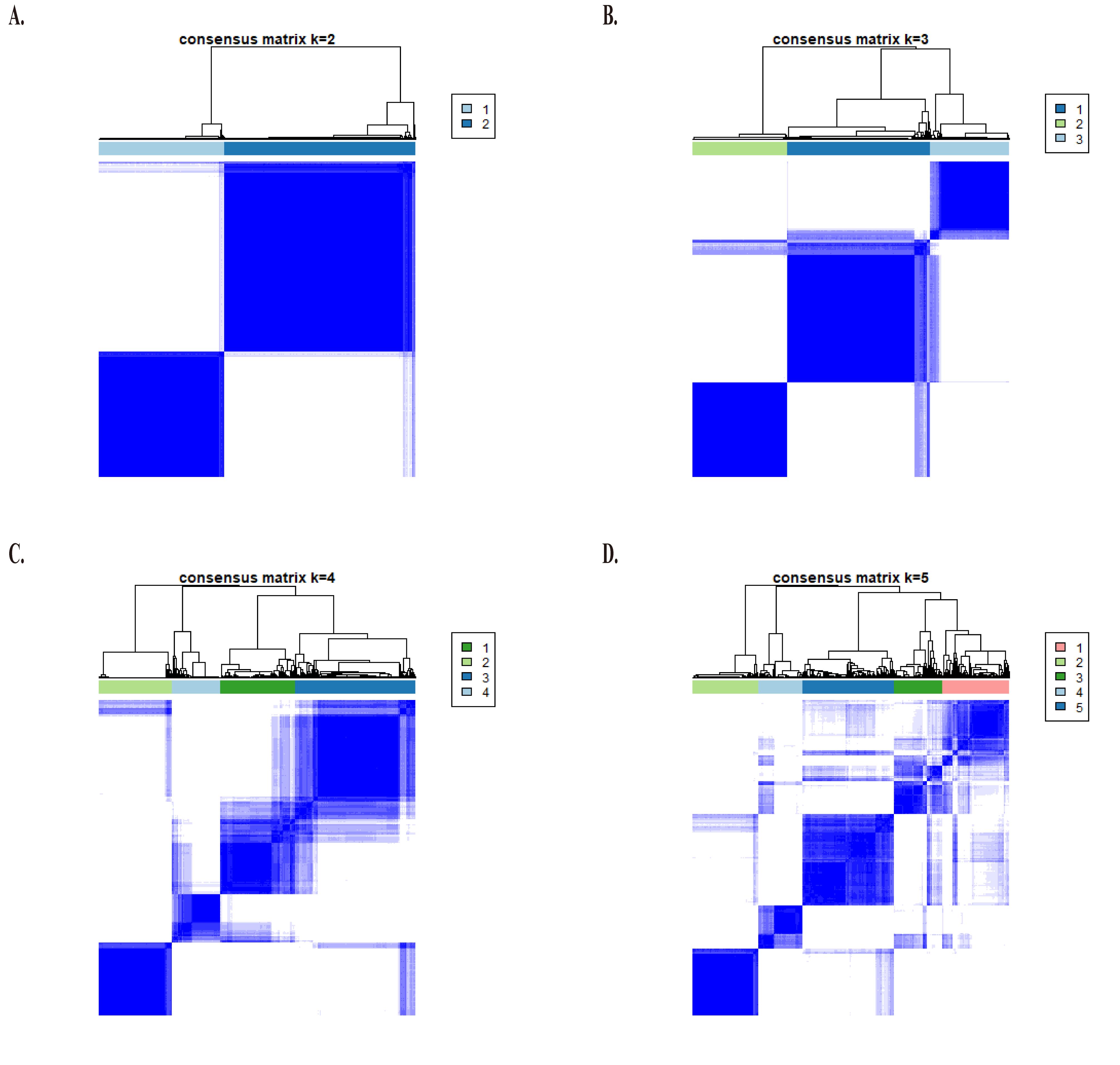

Supplement: Supplementary Figure 4 — (A–D) Consensus clustering matrices of 177 differentially expressed genes in TCGA HNSCC cohort for k = 2-5. [file Image_4.tif]

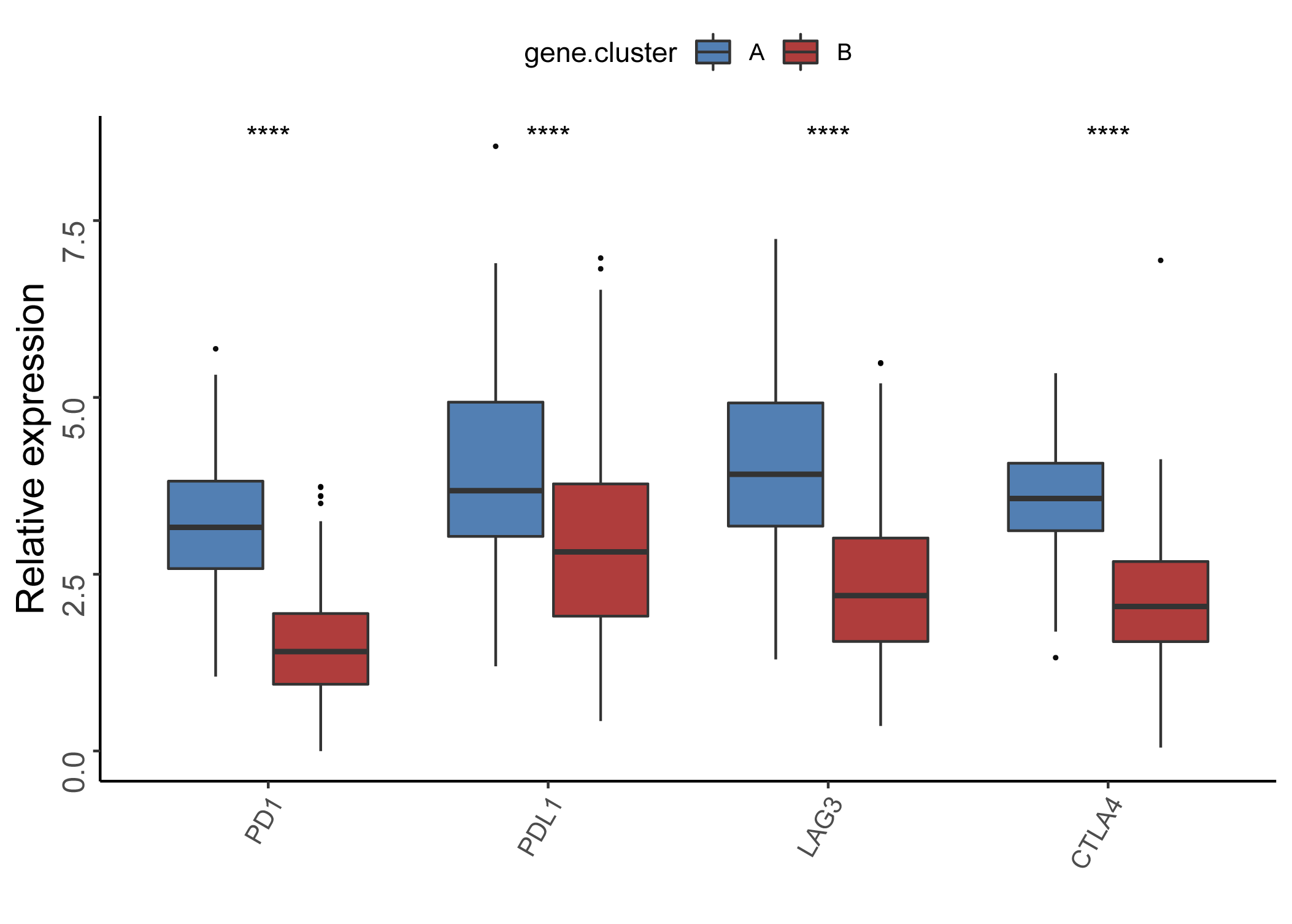

Supplement: Supplementary Figure 5 — The relative expression of PD1, PDL1, LAG3 and CTLA4 in two TNF gene clusters in HNSCC. [file Image_5.tif]

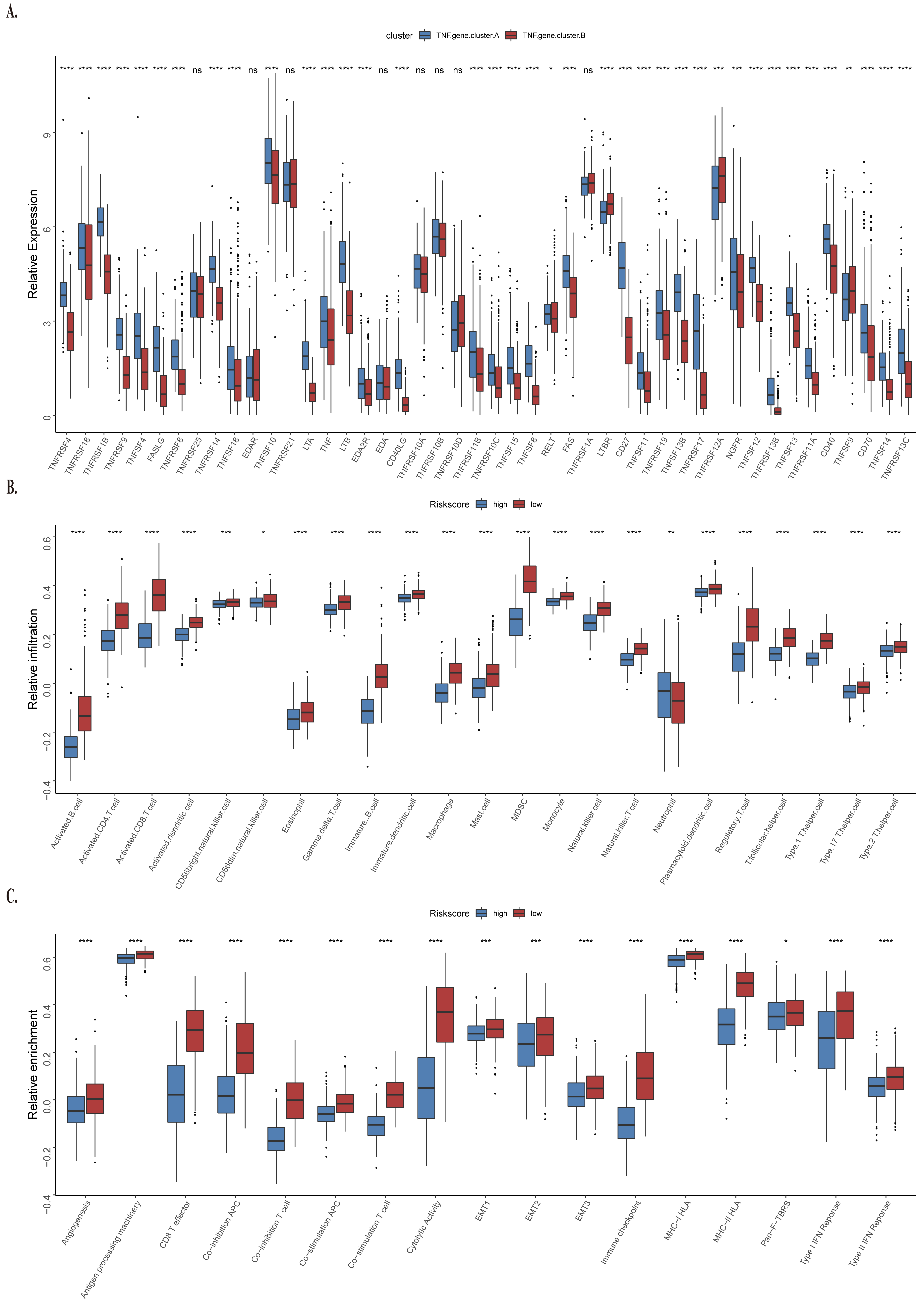

Supplement: Supplementary Figure 6 — (A) Relative expression of 46 TNF family proteins in TNF cluster A and B in TCGA HNSCC cohort. (B) Relative infiltration of 23 types of immune cells in high and low risk group. (C) Relative enrichment score of 17 immune related signatures in high and low risk group. [file Image_6.tif]

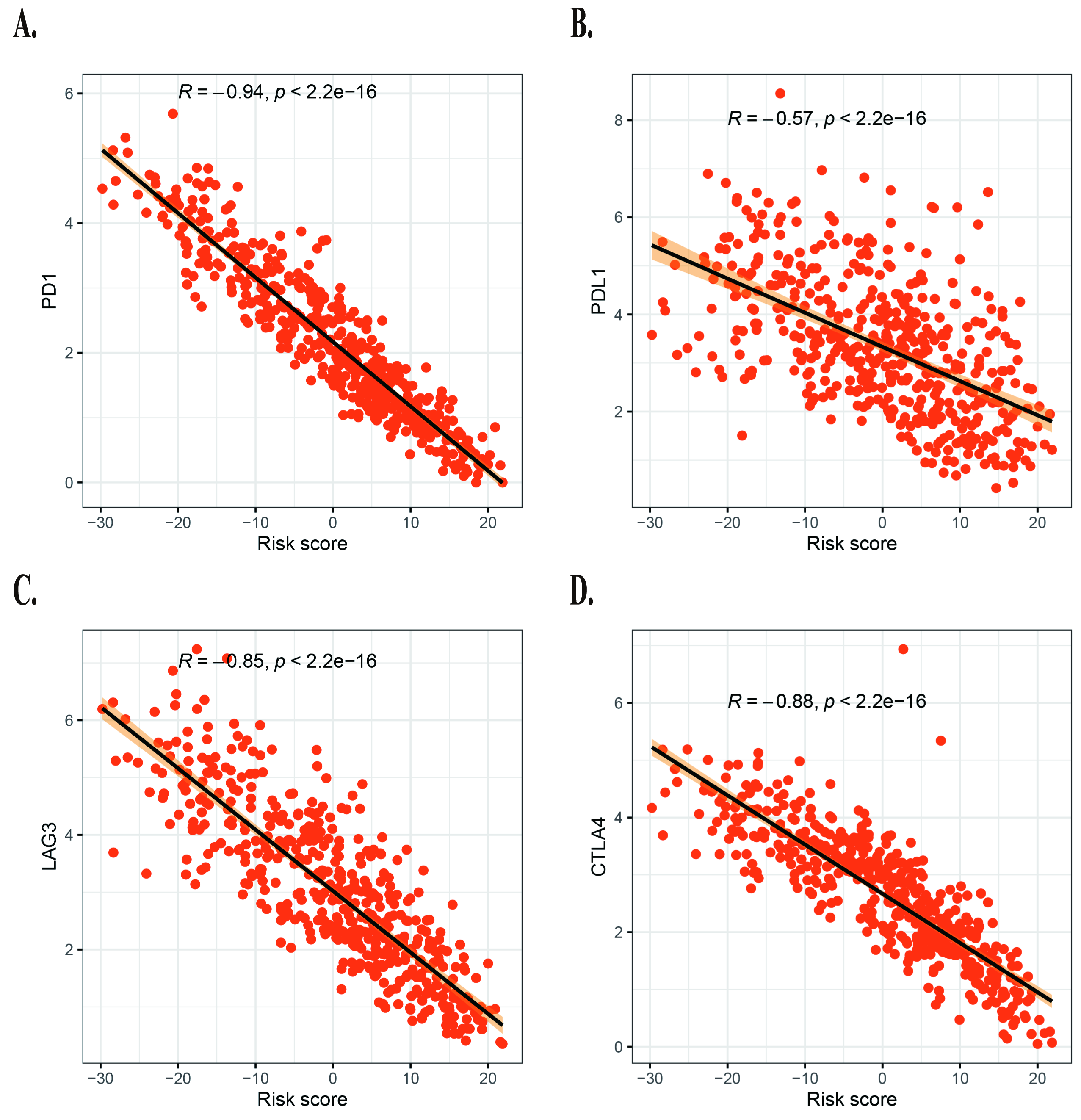

Supplement: Supplementary Figure 7 — (A–D) The correlation of risk score with the expression of PD1, PDL1, LAG3 and CTLA4 in TCGA HNSCC cohort, respectively. [file Image_7.tif]

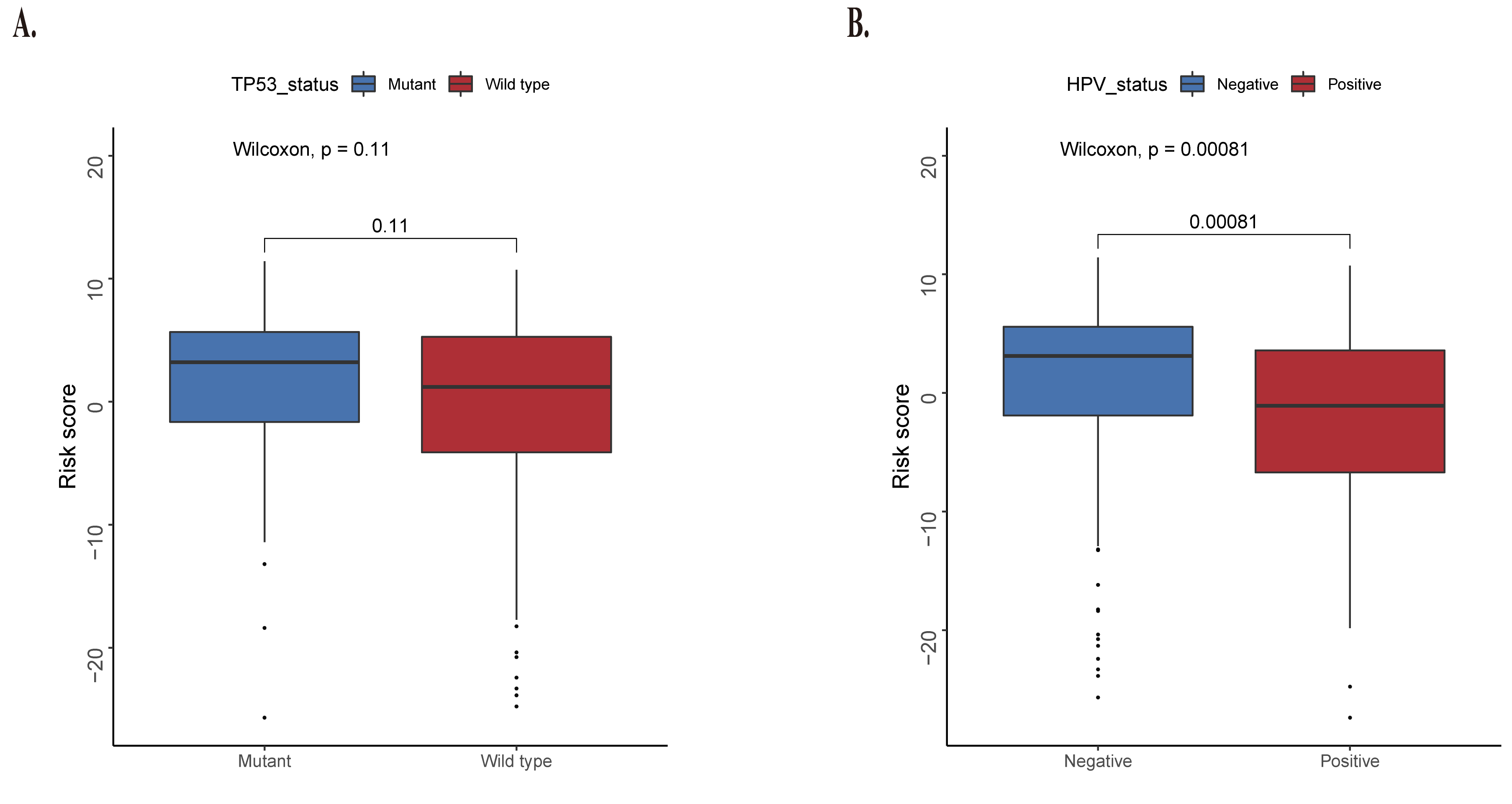

Supplement: Supplementary Figure 8 — (A) Risk score in different TP53 status patients in GSE65858 cohort. (B) Risk score in different HPV status patients in GSE65858 cohort. [file Image_8.tif]
